# A Novel Antidiabetic Drug, Fasiglifam/TAK-875, Acts as an Ago-Allosteric Modulator of FFAR1

**DOI:** 10.1371/journal.pone.0076280

**Published:** 2013-10-10

**Authors:** Chiori Yabuki, Hidetoshi Komatsu, Yoshiyuki Tsujihata, Risa Maeda, Ryo Ito, Kae Matsuda-Nagasumi, Kensuke Sakuma, Kazumasa Miyawaki, Naoya Kikuchi, Koji Takeuchi, Yugo Habata, Masaaki Mori

**Affiliations:** 1 Cardiovascular and Metabolic Drug Discovery Unit, Pharmaceutical Research Division, Takeda Pharmaceutical Company Limited, Fujisawa, Kanagawa, Japan; 2 Central Nervous System Drug Discovery Unit, Pharmaceutical Research Division, Takeda Pharmaceutical Company Limited, Fujisawa, Kanagawa, Japan; CRCHUM-Montreal Diabetes Research Center, Canada

## Abstract

Selective free fatty acid receptor 1 (FFAR1)/GPR40 agonist fasiglifam (TAK-875), an antidiabetic drug under phase 3 development, potentiates insulin secretion in a glucose-dependent manner by activating FFAR1 expressed in pancreatic β cells. Although fasiglifam significantly improved glycemic control in type 2 diabetes patients with a minimum risk of hypoglycemia in a phase 2 study, the precise mechanisms of its potent pharmacological effects are not fully understood. Here we demonstrate that fasiglifam acts as an ago-allosteric modulator with a partial agonistic activity for FFAR1. In both Ca^2+^ influx and insulin secretion assays using cell lines and mouse islets, fasiglifam showed positive cooperativity with the FFAR1 ligand γ-linolenic acid (γ-LA). Augmentation of glucose-induced insulin secretion by fasiglifam, γ-LA, or their combination was completely abolished in pancreatic islets of FFAR1-knockout mice. In diabetic rats, the insulinotropic effect of fasiglifam was suppressed by pharmacological reduction of plasma free fatty acid (FFA) levels using a lipolysis inhibitor, suggesting that fasiglifam potentiates insulin release in conjunction with plasma FFAs *in vivo.* Point mutations of FFAR1 differentially affected Ca^2+^ influx activities of fasiglifam and γ-LA, further indicating that these agonists may bind to distinct binding sites. Our results strongly suggest that fasiglifam is an ago-allosteric modulator of FFAR1 that exerts its effects by acting cooperatively with endogenous plasma FFAs in human patients as well as diabetic animals. These findings contribute to our understanding of fasiglifam as an attractive antidiabetic drug with a novel mechanism of action.

## Introduction

Free fatty acids (FFAs) act not only as a primary energy source in the body but also as cell signaling mediators. Free fatty acid receptor 1 (FFAR1; also known as GPR40) is a G protein-coupled receptor (GPCR) predominantly expressed in human and rodent pancreatic β cells and is activated by physiological concentrations of long- and medium-chain FFAs. Increasing evidence demonstrates that FFAs augment glucose-stimulated insulin secretion (GSIS) in pancreatic β cells by activating FFAR1 [1], [2].

As reported previously, transgenic mice overexpressing human FFAR1 (hFFAR1) in pancreatic β cells exhibit improved glucose excursion and increased insulin secretion during the oral glucose tolerance test (OGTT) and develop resistance to high-fat diet-induced glucose intolerance [3], whereas FFAR1-deficient mice show impaired or unaffected GSIS [4], [5] and absence of insulin and incretin secretion in response to FFAs [6]. These studies clearly identify FFAR1 as an attractive therapeutic target for type 2 diabetes (T2DM).

Fasiglifam (TAK-875), an orally available selective FFAR1 agonist, improves postprandial and fasting hyperglycemia by potentiating GSIS in diabetic rats [7], [8]. Studies in rodent and human β cells have shown that the activation of FFAR1 with fasiglifam and FFAs increases intracellular calcium concentrations ([Ca^2+^]i) in the presence of glucose [9], [10]. The glucose-dependent action of fasiglifam makes it a promising candidate for a novel therapy for diabetes with a low risk of hypoglycemia, in contrast to the widely prescribed antidiabetic drug sulfonylurea, which increases insulin release regardless of glucose levels and can lead to hypoglycemia. Among various FFAR1 agonists described till date [8], [11]–[15], fasiglifam is the most advanced clinical candidate for the treatment of T2DM, showing a potent antidiabetic effect comparable with that of sulfonylureas in early-stage clinical trials, with a lower propensity to cause hypoglycemia in healthy volunteers and diabetic patients [16]–[19]. However, the precise mechanism of the potent pharmacological effect of fasiglifam is not fully understood.

Here we demonstrate that fasiglifam is an ago-allosteric modulator of FFAR1, which amplifies the agonistic activity of the endogenous ligand γ-linolenic acid (γ-LA) by binding to an allosteric site of FFAR1. Fasiglifam alone exhibited partial agonistic activity in cells expressing moderate levels of FFAR1, and exerted positive cooperative effects with FFAs *in vitro* and *in vivo*. Our results provide insight into the mechanism underlying pharmaceutical benefits of fasiglifam as a novel agent for the treatment of T2DM.

## Results

### Fasiglifam Exerts Partial Agonistic Activity for hFFAR1 and mFFAR1

**Figure 1:**
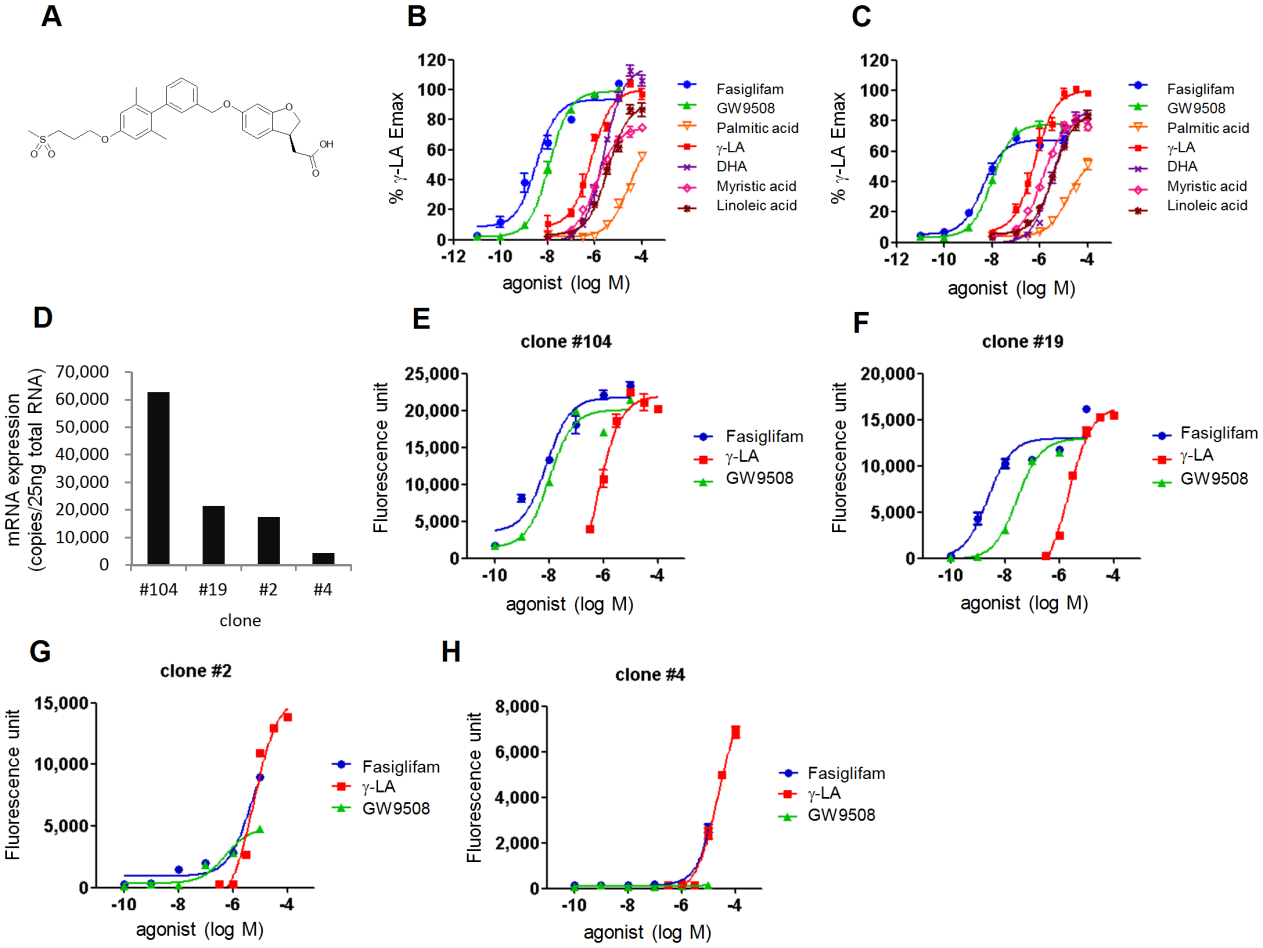
Partial agonist activity of fasiglifam is affected by FFAR1/GPR40 expression levels. (*A*) The chemical structure of fasiglifam. (*B* and *C*) FFAR1 agonist activities of fasiglifam and free fatty acids (FFAs) in the intracellular Ca^2+^ mobilization assay using CHO cell lines expressing hFFAR1 (clone #104) (*B*) or mFFAR1 (*C*). Data are representative of three experiments. (*D*) hFFAR1 mRNA levels of hFFAR1-expressing CHO clones were evaluated by qRT-PCR. (*E* **-** *H*) Relative Ca^2+^ influx activities of γ-LA and fasiglifam in CHO clones #104 (*E*), #19 (*F*), #2 (*G*), and #4 (*H*) with various hFFAR1 expression levels. Error bars indicate s.e.m. (n = 3).

FFAR1 couples with Gαq and induces rapid increase in [Ca^2+^]i by its activation. To investigate the pharmacological properties of fasiglifam and its functional relationships with endogenous ligands, agonistic activities of fasiglifam, various FFAs, and the FFAR1/FFAR4 (GPR120) dual agonist GW9508 [11] were evaluated using a Ca^2+^ mobilization assay in Chinese hamster ovary (CHO) cells stably expressing hFFAR1 or mouse FFAR1 (hFFAR1/CHO or mFFAR1/CHO). In hFFAR1/CHO cells, fasiglifam activated Ca^2+^ influx with higher potency than FFAs (Figure 1B and Figure S1). Interestingly, palmitic acid and myristic acid exhibited partial activities, whereas γ-LA showed full agonistic activity with relatively high potency and efficacy levels among the FFAs tested (Figure 1B and Table S1). In addition, fasiglifam showed lower E_max_ than γ-LA in mFFAR1/CHO cells (Figure 1C and Table S1), suggesting that fasiglifam is a partial agonist for mFFAR1. Because the efficacies of partial agonists depend on receptor expression levels [20], we speculated that fasiglifam may also act as a partial agonist for hFFAR1 in cells with lower receptor expression. Thus, we established hFFAR1-expressing CHO cell lines with different receptor mRNA expression levels (clones #104, #19, #2, and #4) (Figure 1D). In a Ca^2+^ mobilization assay, relative efficacies of fasiglifam varied among the clones and correlated with hFFAR1 mRNA expression levels (Figure 1E-H). To confirm partial agonism of fasiglifam, human embryonic kidney (HEK) 293T cells transiently transfected with varying amounts of hFFAR1 expression plasmids were also used. We found that as the amount of transfection plasmids decreased, maximal responses of fasiglifam dramatically decreased compared with those of γ-LA (Figure S2). These results clearly indicate that fasiglifam exerts partial agonistic activity against both hFFAR1 and mFFAR1.

### Fasiglifam Acts as an Ago-allosteric Modulator of FFAR1 and shows Positive Cooperativity with FFA

**Figure 2:**
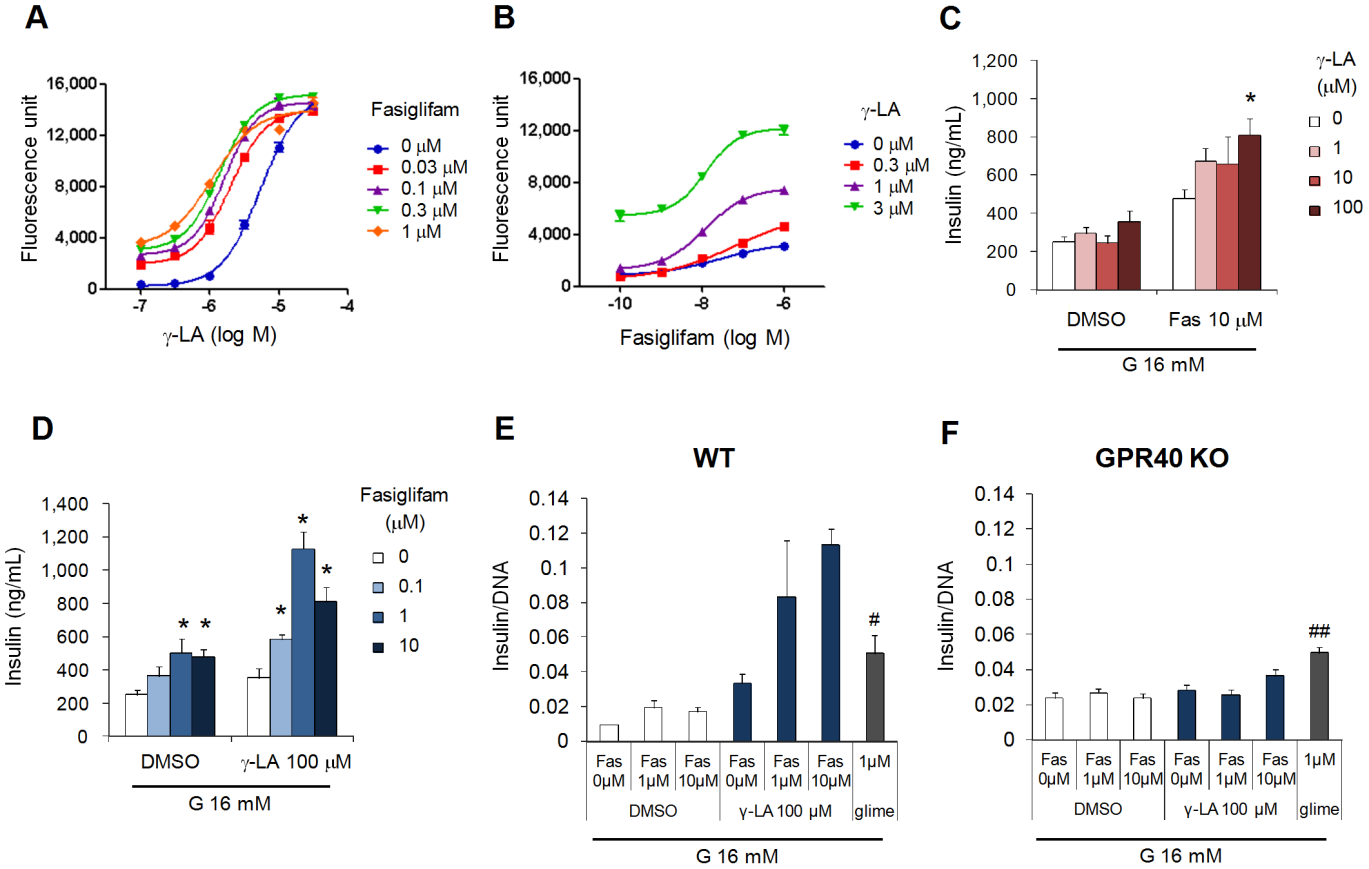
Fasiglifam exhibits positive cooperativity with γ-LA in intracellular Ca^2+^ influx and insulin secretion. (*A* and *B*) Allosteric modulation of γ-linolenic acid (γ-LA) activity by fasiglifam (*A*) and fasiglifam activity by γ-LA (*B*) in the Ca^2+^ mobilization assay using hFFAR1/GPR40-expressing CHO cells (clone #2). (*C* and *D*) Positive cooperative effect of fasiglifam (Fas) on γ-LA activity (*C*) and of γ-LA on fasiglifam activity (*D*) in insulin secretion in mouse pancreatic β cell line, MIN6 cells. (*E* and *F*) Fasiglifam-induced insulin secretion in the absence and presence of γ-LA in pancreatic islets of wild-type (*E*) and FFAR1-knockout mice (*F*). All insulin secretion assays (*C*-*F*) were conducted in the presence of 16 mM glucose (G). All data are representative of at least two replicates. Error bars indicate s.e.m. (n = 3). *P<0.025 by one-tailed Shirley-Williams test, ^#^ *P*<0.05, ^##^ *P*<0.01 versus DMSO alone by Student’s t-test.

In spite of its partial agonist activity *in vitro*, preclinical and clinical trial data demonstrate potent insulinotropic and hypoglycemic effects of fasiglifam in T2DM animal models and diabetic patients [8], [16]. To clarify the interaction between fasiglifam and endogenous FFAs upon activation of FFAR1 and subsequent insulin release, we investigated how the agonist activity of γ-LA is affected by the presence of fasiglifam or *vice versa*. For Ca^2+^ influx analysis, we used hFFAR1/CHO clone #2 because the ratio of fasiglifam and γ-LA activities in this clone was similar to that observed in the mouse pancreatic β cell line MIN6 expressing endogenous FFAR1 (data not shown). Marked shift in the dose–response curve of γ-LA was observed upon addition of fasiglifam, indicating positive allosteric modulation of γ-LA activity by this drug (Figure 2A). EC_50_ values of γ-LA response decreased from 5.39 µM to 1.07 µM in the presence of 1 µM of fasiglifam (Figure 2A). Conversely, remarkable potentiation of the partial activity of fasiglifam was observed with simultaneous stimulation by increasing doses of γ-LA (Figure 2B).

Following this, we examined the insulinotropic effects of fasiglifam, FFAs, and their combination in MIN6 cells and mouse islets. In MIN6 cells, γ-LA alone could not significantly potentiate insulin secretion (Figure 2C), probably because most γ-LA may have been absorbed by serum albumin included in the assay medium [1]. However, in combination with fasiglifam, insulin secretion was augmented in relation to γ-LA concentration (Figure 2C). Similarly, synergistic potentiation of fasiglifam-stimulated insulin secretion by γ-LA (100 µM) was observed, with an increase in the maximal response of fasiglifam by approximately 1.5–2 fold (Figure 2D). Furthermore, dramatic improvement in the insulinotropic activity of fasiglifam by γ-LA in wild-type mouse islets was completely abolished in FFAR1-knockout mouse islets, while the effect of a sulfonylurea, glimepiride, was not affected (Figure 2E, F). These results strongly indicate that fasiglifam and FFAs synergistically contribute to insulin secretion in pancreatic islets through direct activation of FFAR1.

### Fasiglifam Potentiates Insulin Release Cooperatively with Plasma FFAs *in vivo*

**Figure 3:**
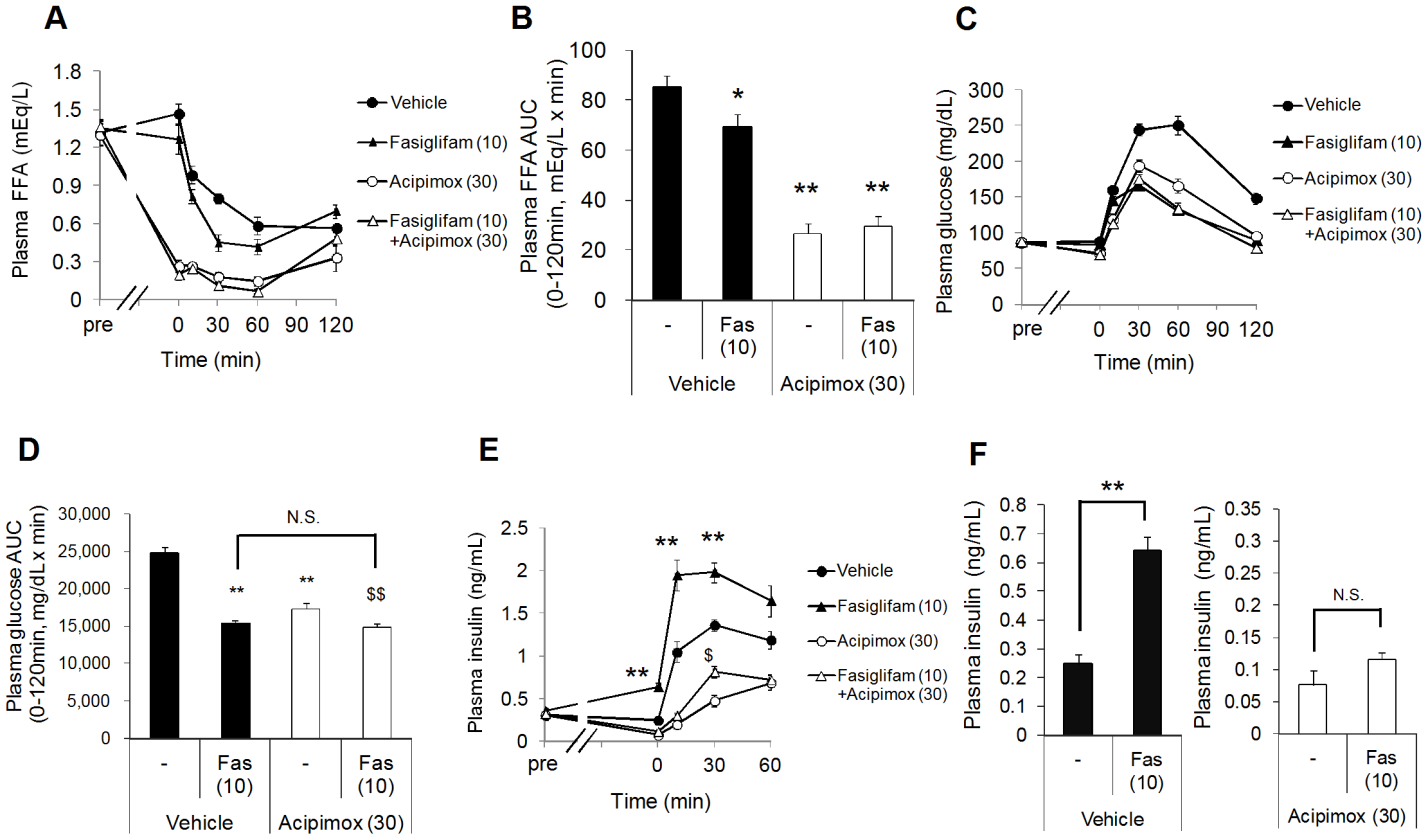
Insulinotropic effects of fasiglifam are attenuated by pharmacological reduction of plasma FFA levels *in vivo*. (*A*) Effects of the lipolysis inhibitors acipimox (30 mg/kg) and fasiglifam (10 mg/kg) on plasma free fatty acids (FFAs) during the oral glucose tolerance test (OGTT) in N-STZ-1.5 rats. (*B*) Area under the curve (AUC) of plasma FFA during 0–120 min. Fas, fasiglifam. (*C*) Plasma glucose levels after coadministration of acipimox (30 mg/kg) and fasiglifam (10 mg/kg). (*D*) AUC of plasma glucose levels during 0–120 min. *P<0.05, **P<0.01 versus vehicle by Student’s t-test, ^$$^ *P*<0.01 versus vehicle by Aspin–Welch test. (*E*) Plasma insulin concentrations after coadministration of acipimox and fasiglifam during OGTT. (*F*) Insulinotropic effects of fasiglifam (Fas) just before glucose load (time 0) shown in (*E*) in the absence and presence of acipimox. **P<0.01 versus vehicle, ^$^ *P*<0.05 versus acipimox alone by Student’s t-test, followed by Bonferroni’s correction for four time point comparisons. Data represent mean ± s.e.m. (n = 6).

To further confirm the physiological relevance of the allosteric interaction between fasiglifam and endogenous FFAs *in vivo*, we conducted OGTT in diabetic neonatal streptozotocin (N-STZ)-1.5 rats, a T2DM model with impaired insulin secretion, under treatment with the lipolysis inhibitor acipimox that lowers plasma FFA levels by inhibiting hormone-sensitive lipase. A rapid decrease in plasma FFA levels was detected with acipimox (30 mg/kg) treatment in N-STZ-1.5 rats (Figure 3A), with a significant reduction of the area under the curve (0–120 min; Figure 3B). Acipimox alone improved glucose tolerance to the level similar to those seen with fasiglifam alone and fasiglifam+acipimox (Figure 3C, D), possibly because of acute reduction of plasma lipid levels, which leads to improved insulin sensitivity as shown in humans [21], [22]. These conditions seemed to be suitable for evaluating the effects of plasma FFA levels on the insulinotropic action of fasiglifam because blood glucose concentrations, which may affect insulinotropic effects of fasiglifam, were similar between fasiglifam alone and fasiglifam+acipimox treated groups during OGTT. In rats treated with fasiglifam alone, significant insulinotropic effects were observed at 0, 10, and 30 min (Figure 3E, black triangle) compared to vehicle treated rats. In the acipimox treated groups, the increase in plasma insulin after glucose stimulation was much smaller, probably because of decreased enhancement of GSIS by plasma FFAs [23], [24] and/or rapid improvement in insulin sensitivity [21] (Figure 3E, white circle and white triangle). Under such conditions, the insulinotropic action of fasiglifam was significantly suppressed in acipimox-treated rats (Figure 3E, white triangle). In particular, augmentation of insulin levels by fasiglifam just before glucose load (0 min) clearly differed in the absence and presence of acipimox (Figure 3F). Taken together, these observations indicate that endogenous plasma FFAs play an important role in the insulinotropic action of fasiglifam in the rodent model of T2DM, supporting the results of *in vitro* analysis. Our results may also provide mechanistic insights into the potent drug efficacy of fasiglifam in human diabetic patients.

### Fasiglifam does not Exacerbate Fatty Acid-induced Lipotoxicity in β cells

**Figure 4:**
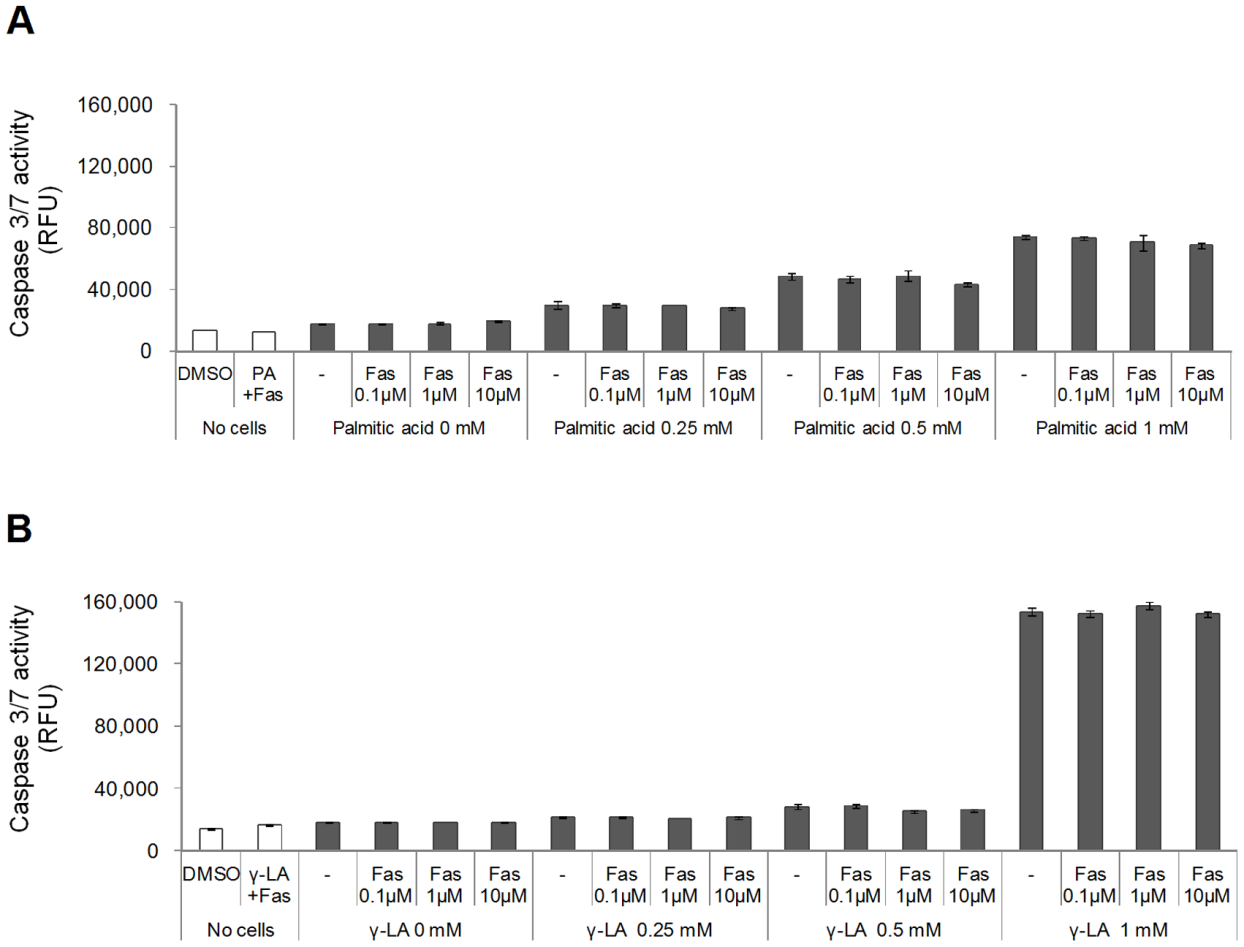
Fasiglifam does not exacerbate FFA-induced apoptotic signaling in MIN6 cells. Caspase 3/7 activity in the mouse pancreatic β cell line MIN6 after 72-h exposure to 0.25–1 mM palmitic acid (*A*) or γ-linolenic acid (γ-LA) (*B*) in combination with fasiglifam (Fas, 0.1–10 µM). “PA+Fas” and “γ-LA+Fas” indicate “1 mM palmitic acid +10 µM fasiglifam” and “1 mM γ-LA +10 µM fasiglifam”, respectively. Data shown are mean ± s.e.m. (n = 3).

Long term exposure of pancreatic β cells to FFAs impairs β cell function and leads to cell apoptosis, an effect known as lipotoxicity [25], [26]. We have previously shown that prolonged exposure to fasiglifam alone had no effect on apoptosis in rat insulinoma cells [8], consistent with recent reports suggesting little involvement of FFAR1 in the mechanism of lipotoxicity [3], [4], [27], [28]. To further confirm that fasiglifam does not enhance β cell toxicity of FFAs, we examined the effect of fasiglifam on FFA-induced caspase activation in MIN6 cells. Seventy-two-hour exposure of MIN6 cells to palmitic acid (0.25–1 mM) increased caspase 3/7 activity in a dose-dependent manner (Figure 4A). Treatment with γ-LA showed weaker cell toxicity; however, high concentrations (1 mM) of γ-LA caused significant enhancements of caspase 3/7 activity (Figure 4B). As expected, the addition of fasiglifam did not further enhance the lipotoxic effects of either FFA at any concentration (Figure 4A, B). This observation strongly suggests that FFAR1 is not involved in FFA-induced lipotoxicity and that fasiglifam does not exacerbate these toxic effects.

### Mutations of FFAR1 Differentially Affect Receptor Activation by Fasiglifam and γ-LA

**Figure 5:**
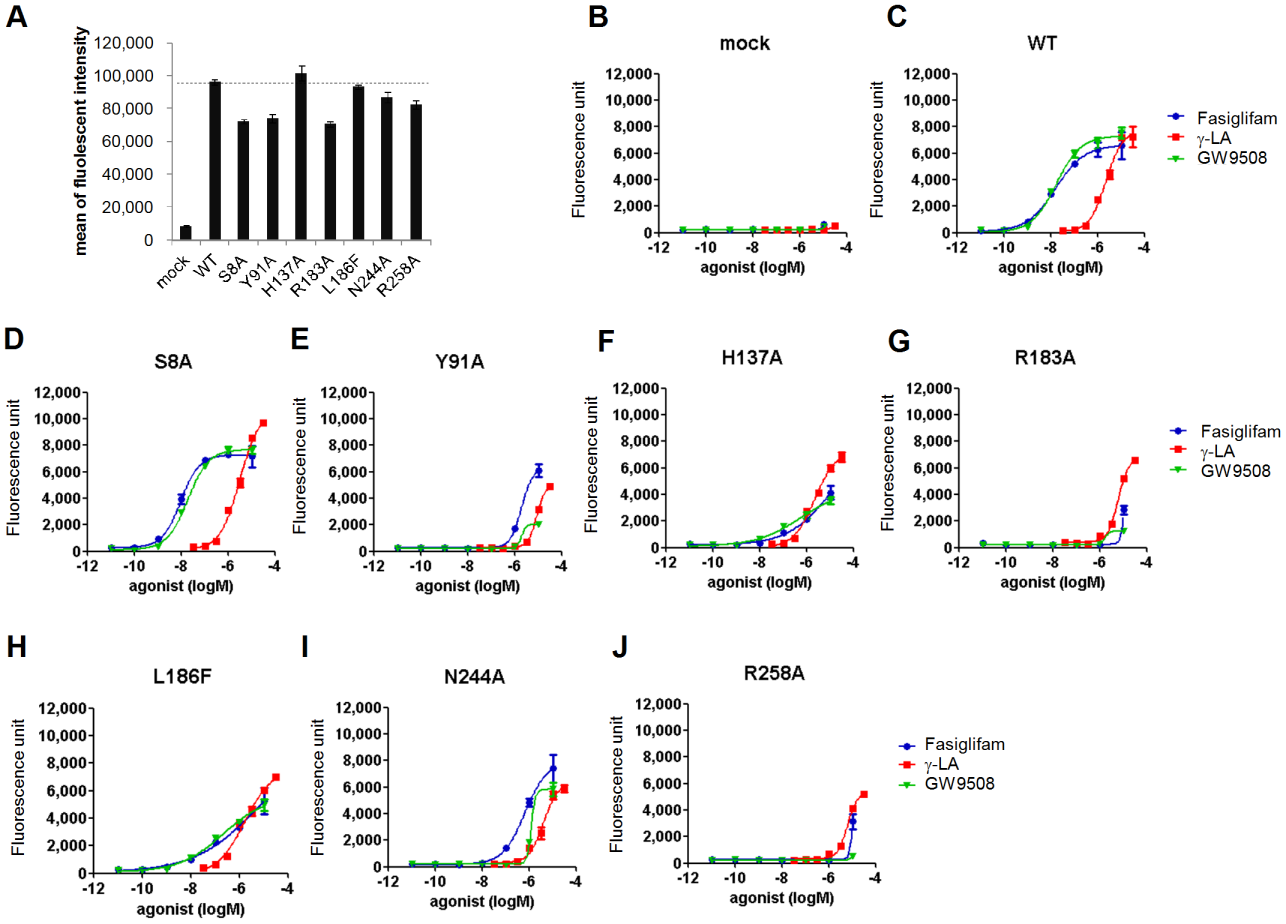
Point mutations of FFAR1/GPR40 differentially affect Ca^2+^ influx activities of fasiglifam and γ-LA. (*A*) Relative cell surface expression levels of FLAG-tagged FFAR1 wild**-**type and mutant receptors in transfected HEK293T cells were determined using flow cytometric analysis (FACS). (*B* **-** *J*) Effects of FFAR1 point mutations on the Ca^2+^ influx activities of FFAR1 agonists. HEK293T cells were transiently transfected with mock vector (*B*), wild-type (*C*), S8A (*D*), Y91A (*E*), H137A (*F*), R183A (*G*), L186F (*H*), N244A (*I*), and R258A (*J*) constructs. Data are representative of three independent experiments. Error bars indicate s.e.m. (n = 3); γ-LA, γ-linolenic acid.

Positive cooperativity of fasiglifam and γ-LA both *in vitro* and *in vivo* indicated that these agonists may bind to distinct sites of FFAR1. To investigate the difference in amino acid residues of FFAR1 involved in the binding and activation of fasiglifam and γ-LA, we examined the effects of seven point mutations at residues previously identified to be important for interactions with various FFAR1 agonists [7], [29]–[32] (Figure 5B–J). Flow cytometric analysis with anti-FLAG tag antibody showed comparable cell-surface expression of wild-type and mutant receptors in transfected HEK293T cells (Figure 5A). In a Ca^2+^ influx assay, R183A and R258A mutants were almost unresponsive to fasiglifam (Figure 5G, J and Table S2), consistent with the literature demonstrating the key roles of these positively charged/hydrophilic residues in the binding of carboxyl groups of FFAR1 agonists [29]. In comparison, the potencies of γ-LA were relatively less affected by these mutations, suggesting that Arg183 and Arg258 are important residues for both fasiglifam- and γ-LA-induced activation, but to a greater extent for fasiglifam. Similarly, the activities of all agonists were affected by Y91A and N244A mutations; however, the effects were more drastic on the potencies of fasiglifam and GW9508 (Figure 5E, I and Table S2). Reportedly, His137 and Leu186 affect the binding of GW9508 but not linoleic acids [29]. Indeed, these residues appeared to be important for the recognition of fasiglifam and GW9508 because the EC_50_ values of these compounds were decreased by 7.7- to 130-fold, while γ-LA activity remained almost unchanged in H137A and L186A mutants (Figure 5F, H and Table S2). The S8A mutation, localized in transmembrane domain 1, had little effect on any of the agonist responses (Figure 5D). Differing patterns of mutational effects on fasiglifam and γ-LA activities strongly support our finding from functional studies that these agonists bind to distinct binding sites of FFAR1, followed by a cooperative activation of this receptor. Mutational analysis also revealed that fasiglifam and GW9508 exert similar binding modes against FFAR1.

## Discussion

**Figure 6:**
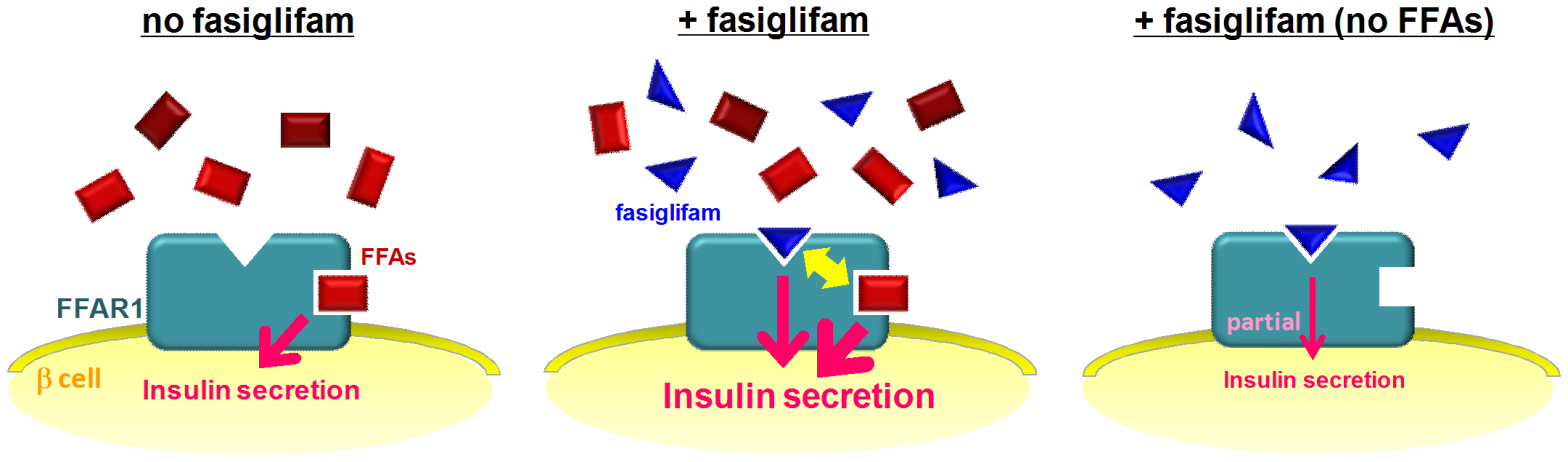
Schematic model of insulinotropic action of fasiglifam in cooperativity with endogenous FFAs in β cells FFAR1 activation by endogenous FFAs contributes to glucose-stimulated insulin secretion (GSIS) *in vivo* (*left*). Fasiglifam treatment potentiates FFA-induced insulin secretion as an allosteric modulator, whereas FFAs also augment the activity of fasiglifam, showing reciprocal positive cooperativity (*middle*). Fasiglifam treatment in the absence of FFAs (nonphysiological) results in partial activation of FFAR1 and weak potentiation of GSIS (*right*).

In clinical trials, fasiglifam has been demonstrated to show robust glucose-lowering effects with no severe side effects in T2DM patients [16]. Preclinical studies have also provided evidence of its effectiveness in rodent models [7], [8]. However, mechanistic explanations for the attractive pharmacological features of fasiglifam have not been fully proposed, such as glucose-dependent mechanism of action. It is also unclear how fasiglifam and endogenous ligands in plasma contribute to the overall insulin secretion in pancreatic β cells. In this study, we demonstrated that fasiglifam is an ago-allosteric modulator of FFAR1, which binds to allosteric binding site of FFAR1 and exerts its pharmacological action by activating the receptor in cooperativity with endogenous FFAs (Figure 6). Our results showed that fasiglifam alone exhibits partial agonist activity, and its efficacy was strongly affected by receptor expression levels in the system. In addition, FFAR1-dependent positive cooperative effects between fasiglifam and γ-LA were observed with Ca^2+^ signaling in low receptor-expressing CHO cell line and insulin secretion in pancreatic MIN6 cells or mouse islets. Furthermore, reduction of plasma FFA levels resulted in marked attenuation of the insulinotropic effects of fasiglifam, indicating that mutual potentiation with endogenous ligands is essential for the pharmacological effects of this drug in the rodent model of T2DM. These observations provide insights into the mechanism underlying the potent hypoglycemic effect of fasiglifam in human diabetic patients. To the best of our knowledge, this is the first report to demonstrate the cooperative effect of FFAR1 agonists with endogenous plasma FFAs *in vivo*.

Reduced insulinotropic actions of fasiglifam under low plasma FFA suggest that the efficacy of this drug may be largely affected by the changes in plasma FFA levels during the day. In a previous report, elevated plasma FFA concentrations (0.4–0.8 mEq/L) in T2DM patients were shown throughout a 24-h study, while these remained within the range of 0.2–0.5 mEq/L in normal individuals [33]. Increased plasma FFA in T2DM is likely due to low plasma insulin levels in these patients because plasma insulin is known to lower FFA release from adipose tissue by inhibiting hormone-sensitive lipase [34]. Accordingly, low plasma FFA levels observed after artificial reduction with acipimox in diabetic rats (approximately 0.2 mEq/L) were only observed in limited time periods in normal individuals but not in severe T2DM patients. In this context, fasiglifam may act more effectively under appropriate conditions (i.e., low plasma insulin level and elevated plasma FFA level), whereas its effects are milder when insulin levels are high enough to control plasma FFA as well as plasma glucose below a certain level. This can be another explanation for the low hypoglycemic risk of fasiglifam in addition to its glucose-dependent mechanism of action.

Because fasiglifam was revealed to be an allosteric modulator of FFAR1, the receptor binding modes of fasiglifam and endogenous ligands are of great interest. Several recent homology-modeling and mutational studies of FFAR1 have demonstrated docking models of the receptor in complex with FFAR1 agonists, including fasiglifam, linoleic acid, and GW9508 [7], [29]–[32], [35]. Previous mutational studies have described that the carboxyl groups of linoleic acid and GW9508 bind to FFAR1 by interacting with positively charged/hydrophilic residues such as Arg183, Asp244, and Arg258. Binding of agonists to FFAR1 leads to breakage of two “ionic locks” (Glu145-Arg183 and Glu172-Arg258 interactions), which function as molecular switches for receptor activation [31]. In our experiment, R183A and R258A mutants were almost completely unresponsive to fasiglifam or GW9508 but were reactive to γ-LA with decreased potency. This suggests that these residues are not only essential for the recognition of fasiglifam but also involved in receptor activation. However, given partial agonism of fasiglifam, we speculate that binding of this compound does not completely break the ionic lock but changes the receptor conformation into a state that is more susceptible to full activation by γ-LA. In contrast, mutation of the hydrophobic residues His137 and Leu186 affected the activities of fasiglifam (or GW9508) but not γ-LA, suggesting that these amino acids are directly or indirectly involved in the binding of fasiglifam or in conformational changes induced by fasiglifam.

Very recently, Lin et al. reported that there are at least three allosterically linked binding sites on FFAR1, identifying a specific agonist for each site [36]. One of the agonists, AMG837, is another clinically developed FFAR1 agonist that acts as a partial agonist and displays positive cooperativity with DHA or full agonists in functional assays [13], [36]. These characteristics of AMG837 are similar to those of fasiglifam and GW9508 shown in this report, suggesting that these agonists may bind to the same binding site, distinct from the orthosteric site to which DHA and γ-LA bind. Alternatively, the idea of multiple binding sites on FFAR1 leads us to speculate that there is no particular “orthosteric site,” but different fatty acids may utilize respective binding sites of FFAR1 to amplify the total signaling. Further combination study of various FFAs would be required to clarify how endogenous FFAs affect one another upon FFAR1 activation in pancreatic β cells.

Another interest for the pharmacological effect of fasiglifam may be an extrapancreatic action of the compound. Recent reports have described that FFAR1 is also expressed in enteroendocrine cells and mediates FFA-induced secretion of incretins such as GLP-1 and GIP [6], [37]. Moreover, a potent class of FFAR1 full agonists has also been shown to induce GLP-1 secretion [38]. FFAR1 full agonists present an attractive opportunity to improve both insulin secretion and incretin effect by a single agent, although the unpredictable effects after prolonged administration of this class of agonists remain unclear. Xiong et al. recently demonstrated *in vivo* GLP-1 secretion stimulated by oral administration of corn oil or a combination of full and partial FFAR1 agonists through the activation of FFAR1 expressed in small intestinal cells [37]. The absence of significant GLP-1 secretion following the administration of full or partial agonist alone reported in this study may be due to weak expression of FFAR1 in enteroendocrine L cells. Therefore, we speculate that although basal plasma FFAs and fasiglifam do not efficiently stimulate GLP-1 secretion, fasiglifam may amplify the induction of GLP-1 secretion by exogenously ingested FFAs such as those in corn oil. Indeed, a tendency for fasiglifam-induced increases in total GLP-1 has been reported in a phase 2 study [19]. In addition, we expect that co-administration of fasiglifam with the minimum dose of full agonists may provide greater effects on both insulin and GLP-1 secretion, with a limited possibility of undesirable effects. Further studies are required to clarify how fasiglifam affects GLP-1 secretion induced by endogenous and exogenous FFAs as well as FFAR1 full agonists in an *in vivo* environment.

To date, no severe adverse events have been reported in clinical phase 1 and phase 2 studies of fasiglifam [16], [18], [39]. Our findings that fasiglifam is an ago-allosteric modulator may explain these attractive therapeutic benefits of this compound because allosteric modulators offer several potential advantages with respect to classical orthosteric compound, which include greater subtype selectivity, spatial and temporal selectivity, and/or lower probability of desensitization [40]. Although allosteric modulators for many GPCRs have been described till date [40], only two of these are currently on the market: cinacalcet [a positive allosteric modulator of the calcium-sensing receptor (CaSR)] and maraviroc (a negative allosteric modulator of the chemokine receptor CCR5). It is intriguing in terms of both basic and clinical research that fasiglifam may become the next example of a GPCR allosteric modulator proven to be effective in human patients.

In conclusion, we have characterized the antidiabetic drug candidate fasiglifam as an ago-allosteric modulator of FFAR1 that exerts potent pharmacological effects by acting cooperatively with endogenous FFAs. The nature of allosteric modulators, which offer greater efficacy in the presence of endogenous ligands, higher selectivity, fewer undesirable effects, and/or receptor desensitization [37] may provide further rationale for the use of this drug in the T2DM treatment paradigm.

## Materials and Methods

### Materials

Fasiglifam (TAK-875; [(3*S*)-6-({2′,6′-dimethyl-4′-[3-(methylsulfonyl)propoxy]biphenyl-3-yl} methoxy)-2,3-dihydro-1-benzofuran-3-yl] acetic acid hemihydrate) was synthesized at Takeda Pharmaceutical Company Limited. γ-LA, linoleic acid, docosahexaenoic acid, and acipimox were purchased from Sigma–Aldrich. Myristic acid, palmitic acid, and glimepiride were purchased from Wako. GW9508 was purchased from Calbiochem.

### Constructs

FLAG-tagged FFAR1 was constructed using a two-step procedure. Initially, untagged FFAR1-pcDNA3.1 was amplified using the forward primer (aaataaaagcttatggactacaaggacgacgatgacaaggacctgcccccgcagctct) and the reverse primer (aaataatctagattacttctgggacttgcccc) to insert a FLAG epitope at the N terminus of the receptor. Following this, the polymerase chain reaction (PCR) product was purified and ligated into a pcDNA3.1 vector. Mutations were made using the QuikChange Site-Directed Mutagenesis Kit (Stratagene) and verified by sequencing.

### Cell Lines

To establish CHO cells (dhfr−) stably expressing hFFAR1 or mFFAR1, CHO/dhfr− (ATCC catalog # CRL-9096) cells were transfected with hFFAR1-pAKKO-111H or mFFAR1-pAKKO-111H plasmid using Lipofectamine 2000 (Invitrogen). Cells were selected by culturing in nucleotide-free alpha-minimum essential medium (α-MEM; Invitrogen) supplemented with 10% dialyzed and heat-inactivated fetal bovine serum (FBS; Invitrogen), 100 IU/mL penicillin, and 100 µg/mL streptomycin (Invitrogen). HEK293T (ATCC catalog # CRL-11268) cells were cultured in Dulbecco’s modified Eagle’s medium (DMEM, Invitrogen) supplemented with 10% heat-inactivated FBS, 100 IU/mL penicillin, and 100 µg/mL streptomycin. The murine pancreatic β cell line MIN6 cells [41] were kindly provided by Dr. Miyazaki. MIN6 cells were grown in DMEM containing 10% heat-inactivated FBS, 100 IU/mL penicillin, 100 µg/mL streptomycin, and 55 µmol/L 2-mercaptoethanol (Invitrogen). Cells were cultured in a humidified atmosphere containing 5% CO_2_/95% air at 37°C.

### Ca^2+^ Mobilization Assay using the Fluorometric Imaging Plate Reader System (FLIPR)

For stable CHO cell lines, cells were seeded in black-walled clear-bottomed 96-well cell culture plates (Costar) and incubated overnight in 5% CO_2_ at 37°C. Otherwise, HEK293T cells were transiently transfected with expression plasmids using Trans-IT (Mirus) or Lipofectamine 2000, followed by plating in poly-D-lysine-coated black clear 96-well plates (BD Falcon) and incubated for 2 days. These cells were then incubated at 37°C for 1 h in HEPES-buffered Hank’s balanced salt solution (pH 7.4) containing 2.5 mM probenecid, 1% FBS, and 4 µM fluo-3 AM (Dojindo). Following this, the cells were washed four times with the solution without fluo-3 AM. Intracellular Ca^2+^ concentrations were measured before and after adding samples using FLIPR (Molecular Devices). Assays were performed in the absence of bovine serum albumin (BSA). EC_50_, 95% confidence interval and Emax values for each curve were calculated by data analysis using 4-parameter logistic equation in Prism 5 software (GraphPad Software).

### Quantitative Analysis of hFFAR1 mRNA by Reverse Transcription-PCR (RT-PCR)

Total RNA from CHO cell clones was extracted using ISOGEN (Nippon Gene) according to the manufacturer’s instructions. cDNAs were synthesized from 1 µg of total RNA as per the following procedure: According to the manufacturer’s instructions, the reaction was performed at 42°C using a random primer (Invitrogen) and SuperScriptII reverse transcriptase (Invitrogen). After completion of the reaction, the mixture was diluted to 40 µL with distilled water to prepare cDNA samples of 25 ng template RNA/µL. Taqman RT-PCR was performed using Sequence Detection System Prism 7900 HT (Applied Biosystems) as described previously [42], with primer sets for hFFAR1 (5′-gcccgcttcagcctctct-3′ and 5′-gaggcagcccacgtagca-3′) and fluorescent-labeled probes [5′(FAM)-tctgcccttggccatcacagcct-(TAMRA)3′], respectively. Expression levels were calculated using the standard oligomer (gcccggcccgcttcagcctctctctcctgctcttttttctgcccttggccatcacagccttctgctacgtgggctgcctccgggc) as a template.

### Insulin Secretion Assay

MIN6 cells were seeded at a density of 6 × 10^4^ cells/well in 96-well plates (BD Falcon), and cells were cultured in the growth medium described above for 2 days before experiments. After discarding the medium, cells were preincubated for 2 h at 37°C with 200 µL of Krebs-Ringer bicarbonate–HEPES (KRBH) buffer (116 mmol/L NaCl, 4.7 mmol/L KCl, 1.17 mmol/L KH_2_PO_4_, 1.17 mmol/L MgSO_4_, 25 mmol/L NaHCO_3_, 2.52 mmol/L CaCl_2_, and 24 mmol/L HEPES) containing 0.2% BSA and 1 mmol/L glucose. After discarding the preincubation buffer, KRBH containing 0.2% BSA, 16 mmol/L glucose, and stimulators as shown was added and the plate was incubated for 2 h at 37°C. After incubation, supernatants from each well were collected and secreted insulin concentrations were measured using alphaLISA (PerkinElmer) according to the manufacturer’s instruction.

### Animals

The care and use of the animals and the experimental protocols used in this research were approved by the Experimental Animal Care and Use Committee of Takeda Pharmaceutical Company Limited, and the Guide for the Care and Use of Laboratory Animals were maintained throughout the study (Institute of Laboratory Animal Resources, National Academic Press 1996; NIH publication number 85–23, revised 1996). Sixteen-week-old male wild type and FFAR1-knockout mice (described previously in reference 5) were obtained from Takeda Rabics Limited. Male N-STZ-1.5 rats were generated by subcutaneous injection of 120 mg/kg STZ in male Wistar Kyoto rats 1–2 days after birth. All the animals were fed regular chow CE-2 (CLEA, Japan) and tap water *ad libitum*, with controlled temperature (23–25°C), humidity (50–60%), and lighting (lights on from 7∶00 to 19∶00).

### Preparation of Mouse Islets

Islets were isolated from mouse pancreata by collagenase digestion [43]. Mice were euthanized by cervical dislocation under sodium pentobarbital anesthesia. A solution of 1 mg/mL collagenase (Wako Pure Chemical Industries) dissolved in Hank’s balanced salt solution (Invitrogen) was injected into the common bile duct. The pancreas was removed and incubated at 37°C for 20 min and washed three times with RPMI 1640 medium containing 5.5 mmol/L glucose and 10% FBS, and islets were hand-collected under a microscope. Freshly purified islets were used for gene expression experiments. For insulin secretion experiments, purified islets were used after overnight culture in RPMI 1640 medium at 37°C, and insulin secreted into culture supernatants was measured as described above. Residual islets were sonicated and used to determine the DNA content (Quant-iT Picogreen dsDNA Assay Kit; Invitrogen).

### Oral Glucose Tolerance Test

Seventeen-week-old N-STZ-1.5 rats were orally administered vehicle (0.5% methylcellulose), fasiglifam (10 mg/kg), acipimox (30 mg/kg), or fasiglifam plus acipimox after an overnight fast. Sixty minutes later, all the animals received an oral glucose load (1.5 g/kg). Blood samples were collected from the tail vein before drug administration (pre), before glucose load (time 0), and 10, 30, 60, and 120 min after the glucose load. Plasma glucose and FFA levels were measured using Autoanalyzer 7080 (Hitachi, Japan), and insulin levels were measured using the Rat Insulin ELISA Kit (Morinaga, Japan). All procedures were conducted according to the Experimental Animal Care and Use Committee of Takeda Pharmaceutical Company Limited.

### Measurement of Caspase 3/7 Activity

To prepare palmitic acid–BSA conjugates, sodium palmitic acid was dissolved in hot distilled water and added to an equal volume of RPMI 1640 medium containing 20% (w/v) FFA-free BSA with stirring on ice. The γ-LA-BSA conjugate was prepared by diluting 500 mM γ-LA in 10% (w/v) FFA-free BSA solution, followed by filtration (0.22 µm). Caspase 3/7 activity in MIN6 cells was measured according to a previously described method (8). In brief, MIN6 cells were seeded at a density of 2 × 10^4^ cells/well in a 96-well black clear plate (CORNING) and cultured overnight. After discarding the medium, 1% BSA, palmitic acid–BSA conjugate (0.25, 0.5, and 1 mM) or γ-LA–BSA conjugate (0.25, 0.5, and 1 mM) in combination with 0.1% DMSO or fasiglifam (0.1, 1, and 10 µM) were added to the plates and cultured for 72 h. After incubation, caspase 3/7 activity was measured using the Apo-one Homogeneous Caspase 3/7 assay (Promega) according to the manufacturers’ instructions. Fluorescent intensity was measured at excitation and emission wavelengths of 485 nm and 535 nm, respectively (Wallac 1420 ARVO SX Multilabel Counter; PerkinElmer).

### Flow Cytometry

HEK293T cells were transiently transfected with wild-type or mutant FFAR1 plasmids as described above and incubated in 5% CO_2_ at 37°C for 2 days. Cells were then collected and stained with Anti-DYKDDDDK (FLAG) tag monoclonal antibody (Wako) for 30 min at room temperature and were then washed two times in phosphate-buffered saline (PBS) containing 2% goat serum. Cells were stained with secondary antibody (Alexa-Fluor 488 goat anti-mouse IgG; Invitrogen) for 30 min, washed two times, and analyzed using Accuri C6 Flow Cytometer (BD Biosciences).

### Data Analysis

Error bars represent the standard error of the mean (s.e.m.). Statistical differences between two groups were analyzed using Student’s t-test or Aspin–Welch test, and dose-dependency was analyzed using one-tailed Shirley-William’s test. In the case of time-dependent changes in insulin, statistical differences were analyzed using Student’s t-test or Aspin–Welch test, followed by Bonferroni’s correction for four time point comparisons.

## Supporting Information

Figure S1 **Intracellular Ca^2+^ increase induced by FFAR1/GPR40 agonists in CHO cells expressing human FFAR1.** Intracellular Ca^2+^ rise after stimulation of γ-linolenic acid (γ-LA) (**A**), palmitic acid (**B**), fasiglifam (**C**), and GW9508 (**D**) was measured in the Ca^2+^ mobilization assay using the hFFAR1-expressing CHO cell line (clone #104). Each trace represents the mean value from the representative assay performed in triplicate.
(TIF)

Figure S2 **Relative efficacy of fasiglifam is affected by FFAR1/GPR40 expression levels in transient HEK293T cells.** Relative Ca^2+^ influx activities of fasiglifam and γ-linolenic acid (γ-LA) were measured in HEK293T cells transiently transfected with 100 ng (**A**), 30 ng (**B**), 10 ng (**C**), 3 ng (**D**), and 1 ng (**E**) of hFFAR1 expression plasmids. Error bars indicate s.e.m. (n = 3).
(TIF)

Table S1 **Ca^2+^ influx activities of FFAR1/GPR40 agonists and free fatty acids in hFFAR1- and mFFAR1-expressing CHO cells.** EC_50_ values were determined in the Ca^2+^ mobilization assay. The log EC_50_ data are mean ± s.e.m. from three independent assays.
(TIF)

Table S2 **Changes in potencies (logEC_50_) of FFAR1 agonists against FFAR1 mutants in comparison with wild-type receptors in Ca^2+^ influx assays.** EC_50_ values were determined in the Ca^2+^ mobilization assay. The log EC_50_ data are mean ± s.e.m. from three independent assays; n.d., not detected.
(TIF)
