# Supplementary figures and images for: A Novel Antidiabetic Drug, Fasiglifam/TAK-875, Acts as an Ago-Allosteric Modulator of FFAR1
Source: PLoS One. 2013 Oct 10;8(10):e76280. doi: 10.1371/journal.pone.0076280 (PMC3794927; doi:10.1371/journal.pone.0076280)

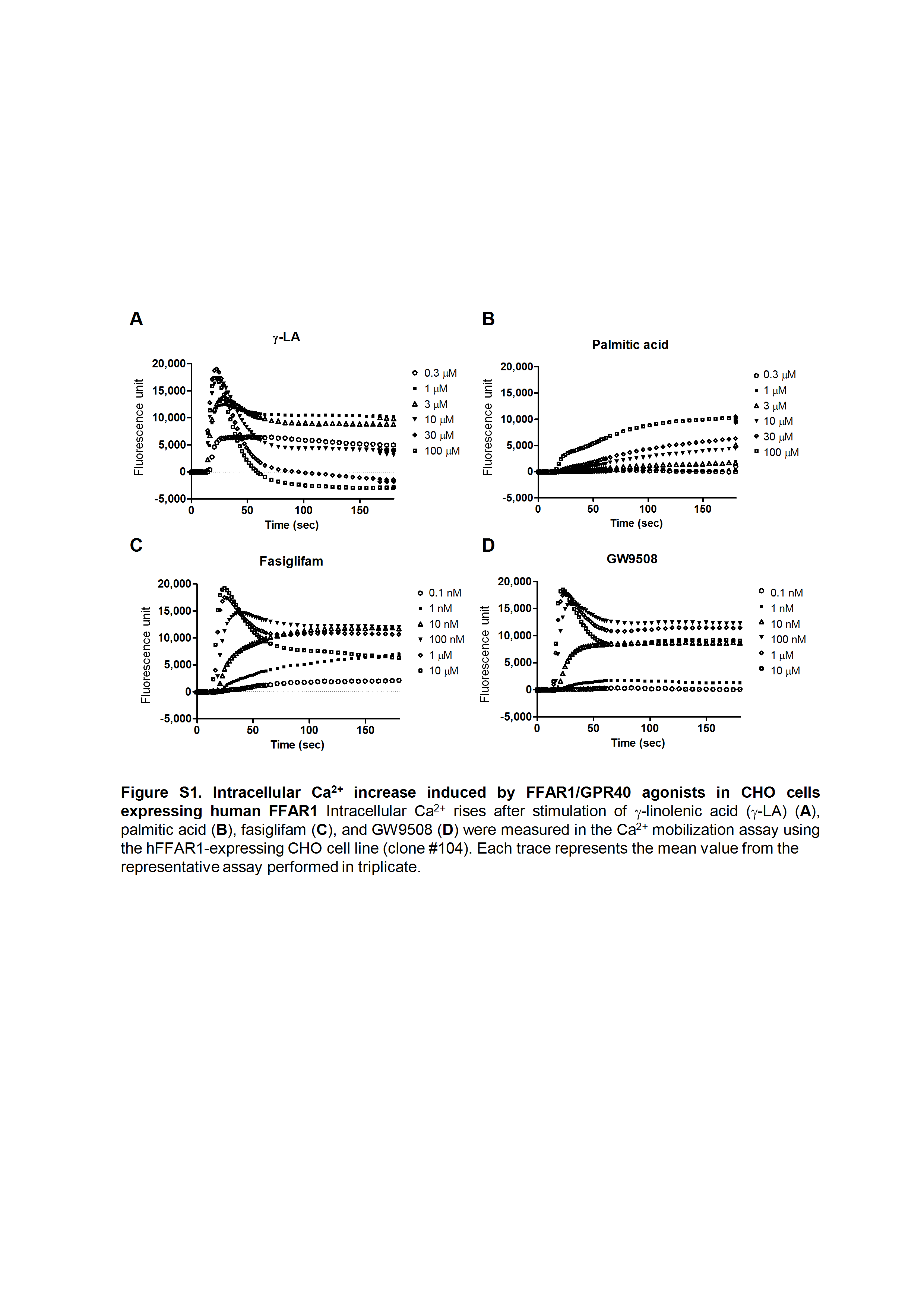

Supplement: Figure S1 — Intracellular Ca2+ increase induced by FFAR1/GPR40 agonists in CHO cells expressing human FFAR1. Intracellular Ca2+ rise after stimulation of γ-linolenic acid (γ-LA) (A), palmitic acid (B), fasiglifam (C), and GW9508 (D) was measured in the Ca2+ mobilization assay using the hFFAR1-expressing CHO cell line (clone #104). Each trace represents the mean value from the representative assay performed in triplicate. (TIF) [file pone.0076280.s001.tif]

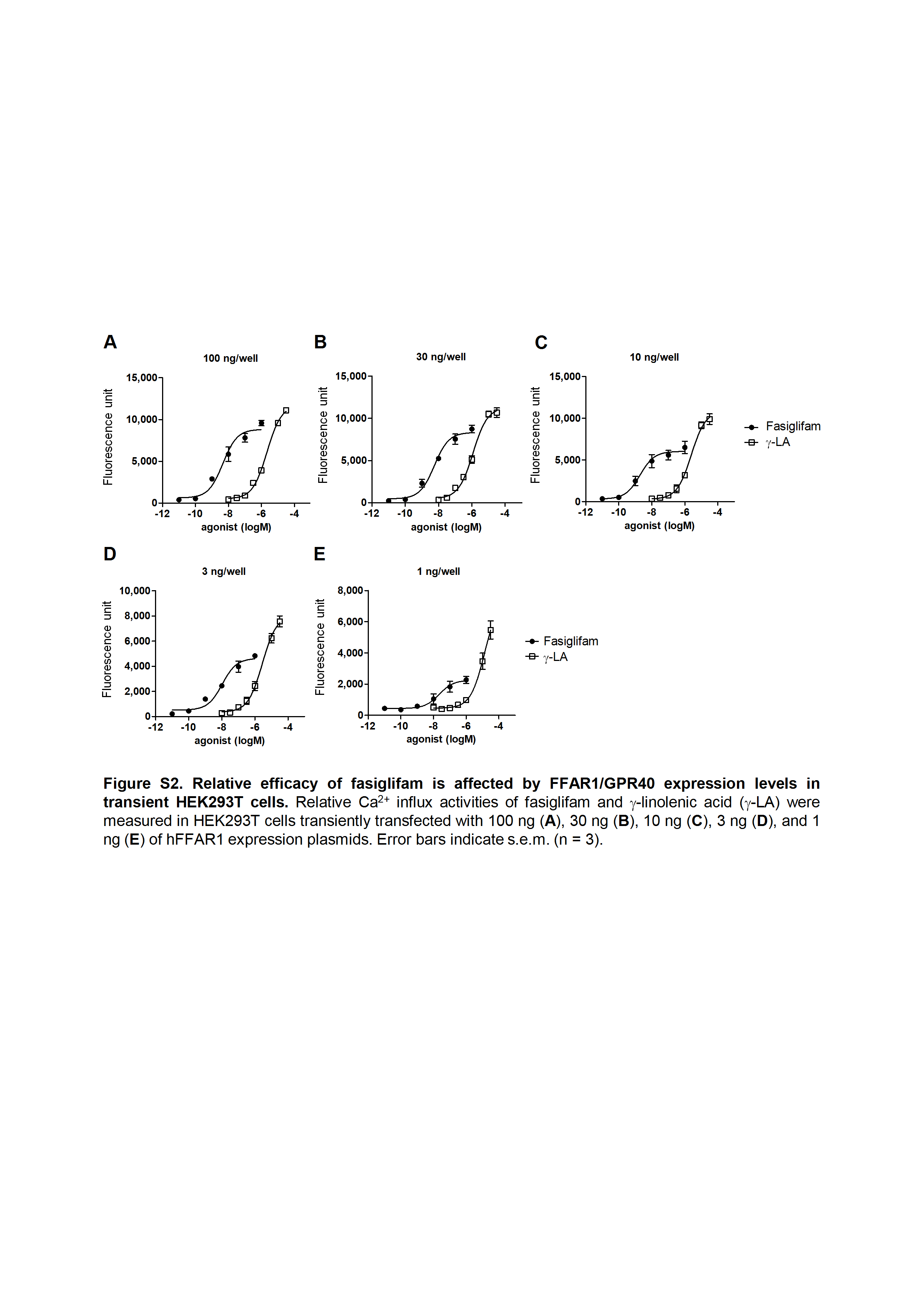

Supplement: Figure S2 — Relative efficacy of fasiglifam is affected by FFAR1/GPR40 expression levels in transient HEK293T cells. Relative Ca2+ influx activities of fasiglifam and γ-linolenic acid (γ-LA) were measured in HEK293T cells transiently transfected with 100 ng (A), 30 ng (B), 10 ng (C), 3 ng (D), and 1 ng (E) of hFFAR1 expression plasmids. Error bars indicate s.e.m. (n = 3). (TIF) [file pone.0076280.s002.tif]

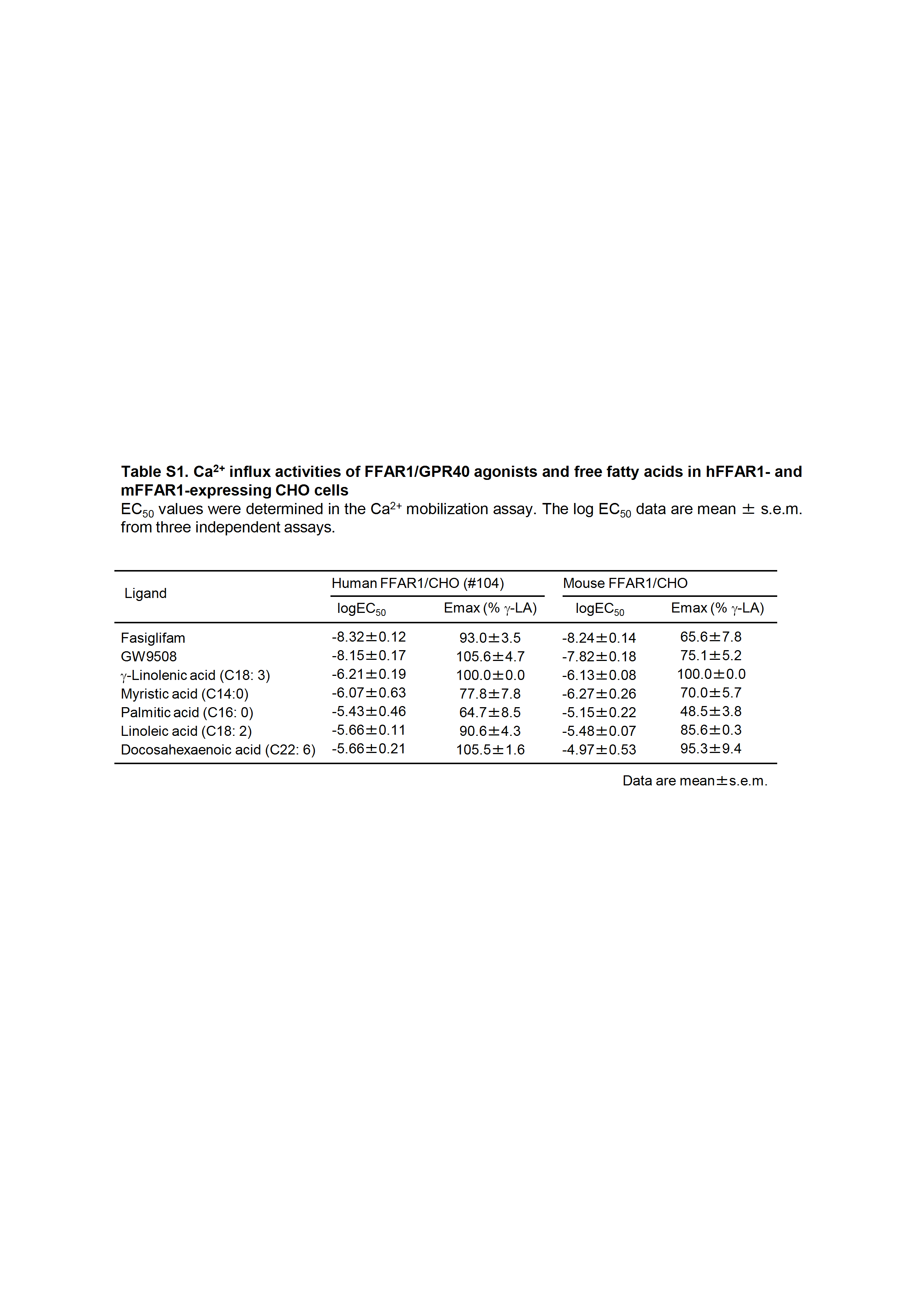

Supplement: Table S1 — Ca2+ influx activities of FFAR1/GPR40 agonists and free fatty acids in hFFAR1- and mFFAR1-expressing CHO cells. EC50 values were determined in the Ca2+ mobilization assay. The log EC50 data are mean ± s.e.m. from three independent assays. (TIF) [file pone.0076280.s003.tif]

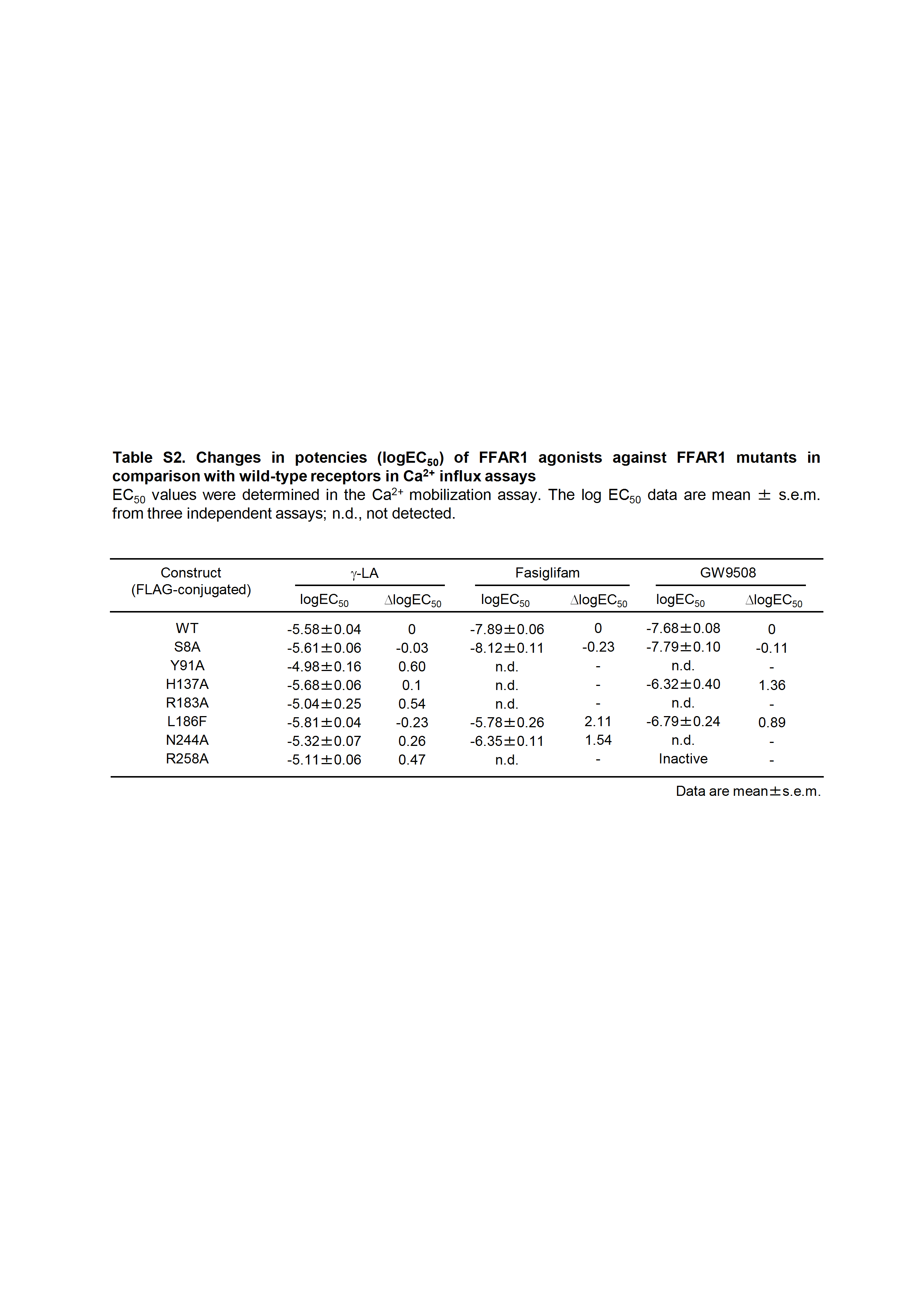

Supplement: Table S2 — Changes in potencies (logEC50) of FFAR1 agonists against FFAR1 mutants in comparison with wild-type receptors in Ca2+ influx assays. EC50 values were determined in the Ca2+ mobilization assay. The log EC50 data are mean ± s.e.m. from three independent assays; n.d., not detected. (TIF) [file pone.0076280.s004.tif]
